# Benefits of Outdoor Sports in Blue Spaces. The Case of School Nautical Activities in Viana do Castelo

**DOI:** 10.3390/ijerph17228470

**Published:** 2020-11-16

**Authors:** Míriam Rocher, Bruno Silva, Gonçalo Cruz, Renato Bentes, Josep Lloret, Eduard Inglés

**Affiliations:** 1 Institut Nacional d’Educació Física de Catalunya (INEFC), Universitat de Barcelona (UB), 08038 Barcelona, Spain; rochermiriam@gmail.com; 2 Escola Superior de Desporto e Lazer de Melgaço, Instituto Politécnico de Viana do Castelo, 320 Melgaço, Portugal; silvabruno@esdl.ipvc.pt; 3 Research Center in Sports Sciences, Health and Human Development (CIDESD), 5001–801 Vila Real, Portugal; 4 Surf Clube de Viana, 4935–161 Viana do Castelo, Portugal; goncalocruz@surfingviana.com (G.C.); renatobentes@surfingviana.com (R.B.); 5 Surfing Viana High Performance Center, 4905–559 Viana do Castelo, Portugal; 6 Oceans and Human Health Chair, Institute of Aquatic Ecology, Universitat de Girona (UdG), 17003 Girona, Spain; josep.lloret@udg.edu; 7 Grup d’Investigació Social i Educativa de l’Activitat Física i l’Esport (GISEAFE, 2017 SGR 1162), Institut Nacional d’Educació Física de Catalunya (INEFC), 08038 Barcelona, Spain

**Keywords:** blue spaces, mental and physical health, outdoor sports, nautical activities, social benefits, health and well-being

## Abstract

Participating in outdoor sports in blue spaces is recognized to produce a range of significant social benefits. This case study empirically analyzes the social benefits associated with the School Nautical Activities project carried out in Viana do Castelo (Portugal) in school-age children and adolescents. It consisted of a 4 year program in which scholars took part in nautical activities (surfing, rowing, sailing, and canoeing) in blue spaces once a week during a semester as a part of their physical education course. The methods used for data collection were as follows: (1) a survey answered by 595 participants in the program and (2) five focus groups (FG): two FGs with participants (seven on each FG), two FGs with their parents (eight participants each), and one FG with the physical education teachers (five participants). Interviews were transcribed and qualitative analysis with NVivo software was developed. Results revealed clear evidence on the social benefits for school-age children and adolescents associated with participation in outdoor activities in blue spaces both in the overall health and in all the following analyzed categories: mental health and well-being, education, active citizenship, social behavior, and environmental awareness. More than 40% state that their overall health is much better now (13.4%) or somewhat better now (29.9%) due to their participation in the program. Thus, this article provides support for the anecdotal recognition of the benefits for school-age children and adolescents from participating in sports in the outdoors and especially in blue spaces.

## 1. Introduction

The time spent in nature and the practice of activities in the outdoors are key determinant factors in promoting human health, preventing disease, and impacting on well-being [1,2,3]. Physical activity (PA) and exercise are also important mediators of health status, with relevant benefits in mortality rates and in preventing or assisting in the treatment of several diseases [4,5]. Several health benefits have been linked to PA by both short-term or long-term studies, such as the increase of blood flow to the brain and neurotransmitter levels [6] or the increased fitness and reduction of blood pressure and obesity [7]. Considering the interaction of these components, participating in outdoor sports can play an important role in human health. Actually, outdoor sports have been shown to successfully break sedentary behaviors, promoting physical quality of life, healthy lifestyle [8], and better stress management and health perceptions [9], with additional positive effects that, by contrast, are not observed when participating in similar physical activity indoors [10]. Being active in nature positively influences cognition [11], educational performance, and motivation [12,13]. Outdoor sports provide unique opportunities within the natural and social environment, often involving the need to work together, leading to intra- and interpersonal development [8,14].

Several studies have associated the practice of outdoor sports with broad categories of benefits such as physical health [15], mental health and well-being [16], education and lifelong learning [14], or community and social behavior [17]. A recent systematic review on the social impacts associated with outdoor sports [8] clusters them into 6 categories: physical health, mental health and well-being, education and lifelong learning, active citizenship, crime reduction, and anti-social behavior; however, the evidence base is not equally strong for all benefits.

Child well-being through PA is associated with playing, particularly playing in nature [18]. Physical education (PE) provides a distinctive opportunity for promoting these benefits, performing an important role in public programs and can be responsible for teaching and promoting a physically active life style in the student community [19]. However, the effects of these activities on children have been hardly investigated [8].

Physical activity and exercise undertaken in natural settings, such as parks and forests (“green spaces”) and rivers, lakes, the coast, and the ocean (“so-called blue spaces,” i.e., environments characterized by the presence of water bodies) are defined as green exercise [20]. There is increasing interest in the benefits of PA in “blue spaces” [21,22], with positive outcomes identified for health and well-being, especially mental health and psycho-social well-being [21]. However, there is a need to research and better understand the role of physical education lessons from a public health perspective [19], particularly the long-term effects of outdoor sports interventions [10] on children and adolescents [8,21]. Some previous works have pointed to the benefits of PA in blue spaces [21,22], but they also state that there is a need of more research in this specific field to prove the real effects not only of the participation in PA in the outdoors but also and concretely in blue spaces.

Therefore, this research aims to address the gap in understanding the impact and social benefits of nautical outdoor sports, as part of PE curriculum, in physical health, mental health and well-being, education and life-long learning, sense of community and active citizenship, social behavior, and environmental awareness in a group of school-age children and adolescents. Conclusions of the above-mentioned previous studies lead us to the assumption that the participation in a nautical outdoor sports program will generate benefits in all the dimensions of the participants’ health.

## 2. Materials and Methods

This descriptive, retrospective study is framed within the post-positivist paradigm and based in a unique case study design: School Nautical Activities project in Viana do Castelo (Portugal).

### 2.1. Setting—The Case of Study

The project School Nautical Activities was developed in Viana do Castelo (Portugal), aiming to bring nautical activities to schools and include them in PE lessons. Coordinated by the city council sports division, school groups from the city of Viana do Castelo participated in surfing, canoeing, rowing, and sailing classes as part of the PE school curriculum. All activities occurred in the municipality’s nautical centers for rowing, canoeing, sailing, and surfing. Participants were all the school-age children from elementary, middle, and high schools. The PE teachers were assisted by specialist instructors in the specific nautical activities, who planned and implemented the programs in accordance with the development of basic and intermediate specific sport skills. All students participated in the activities independent of their sociocultural background, ethnicity, and disability. However, the “Challenge by Choice” principle was applied at all time. Children who have a disability, i.e., autism; motor disability) had an extra teacher, specialized in the area of intervention, who provided assistance in the water for the accomplishment of the adapted learning objectives. The activities took place during a 6 or 12 week mesocycle with a duration of at least 45 min of active time per lesson (excluding travel time and preparations to start the activity).

### 2.2. Inclusion Criteria

Participants who had been enrolled from the beginning of the project were invited to participate in the study.

Thus, inclusion criteria for students to participate in the study were as follows: (i) students currently enrolled in the project, independent of the nautical activity that they were doing at that time; or (ii) students that were enrolled in the project in the last two years, independent of the nautical activity that they had participated in. Moreover, one exclusion criterion was considered: (i) students who were present in the session but never experienced the activity itself.

Hence, all participants who met these criteria were invited to participate in the study in accordance with all ethical standards provided by the Declaration of Helsinki, and the Institutional Review Board thus gave their ethical clearance (CTC-ESDL-CE001-2020).

### 2.3. Instruments

A mixed methodological framework was used, integrating quantitative and qualitative analysis. On the one hand, the instrument used for the quantitative analysis was a survey designed according to the identified categories of the Benefits of Outdoor Sports for Society [8] and carried out with the participants from School Nautical Activities project. On the other hand, qualitative data were gathered through the development of a total of 5 focus groups (FGs). These two instruments and the procedure for data gathering in each case are explained as follows.

#### 2.3.1. Survey

A survey was specifically designed for the study. The selection of items was based on the conclusions of a recent systematic review [8], where a set of dimensions of social benefits of outdoor sports were stated, and complemented by several sources of information to select the most appropriate items, including researchers’ meetings and a panel of experts.

The validation of the survey was developed in two different stages: (i) the first study consisted in the development of a bank of questions to obtain evidence regarding their content validity, response consistency, and comprehensibility; (ii) in the second study, the internal structure was explored and refined using both exploratory and confirmatory factor analysis approaches by the use of the responses to this first version of the survey by a group of 20 participants of a pilot study. The final survey consisted of a total of 42 items assessing five different dimensions: (1) Mental health and well-being (12 items); (2) Education (6 items); (3) Active citizenship (9 items); (4) Preventing anti-social behavior (8 items); and (5) Environment (7 items). All questions, except those regarding type of sports that they participated in and general information/observation, were based on a Likert scale or multiple-choice options, and based on participants’ self-perception.

To accomplish the specifications of the study, a panel of experts in the field reviewed the survey. That group of experts included one sports medicine specialist, one nutritionist, one psychologist, one environment and biodiversity city councilor, one sanitation and sports city councilor, and one sports science and outdoor sports researcher.

*Participants.* From a total sample of 2000 schoolchildren who achieved the inclusion and exclusion criteria, 595 participated in and thus answered the survey. Those were schoolchildren from the schools that facilitated the administration of the survey during one of their PE lessons. Hence, the sample was composed of 595 students participating in the School Nautical Activities (263 males and 330 females, 2 did not indicate their gender; mean age (M) = 14.02 years, standard deviation (SD) = 1.46; range = 11–21 years) attending rural (69.6%, *n* = 414) or non-rural schools (30.4%, *n* = 180). Students participated in several sports during the program, including surfing (65.0%, *n* = 386), rowing (45.8%, *n* = 272), sailing (42.9%, *n* = 255), and canoeing (62.6%, *n* = 372). Participants had been participating in the School Nautical Activities for an average of 2.38 years (SD = 0.95).

*Procedure*. The survey was administered to participants at school, before the start of one of their PE lessons, in their classroom, in a calm and known environment and with the presence of the PE teacher and a member of the research team. The researcher started by explaining the purpose of the study and the associated ethical and logistical issues. Afterward, paper copies of the survey were distributed to each participant. Data collection took place between January and March 2019. All responses were anonymous and confidential, and it took the participants an average of 13 min to complete the survey. All statistical analyses from surveys were completed using SPSS software version 25.0 for MAC (IBM, USA), for a *p* < 0.05. First, preliminary analyses included the detection of missing values and the analysis of data distribution. Then, we calculated Spearman’s rho correlation coefficients between the different dimensions of benefits. Such correlations were interpreted according to the following criteria [23]: trivial (<0.1), small (0.1 ≤ 0.3), moderate (0.3 ≤ 0.5), large (0.5 ≤ 0.7), very large (0.7 ≤ 0.9), and nearly perfect (r ≥ 0.9).

#### 2.3.2. Focus Group

A total of 5 FGs were conducted. The discussion among the participants in each FG was facilitated by the use of an explicitly designed interview guide with several group dynamics. The purpose of the dynamics was to achieve a climate of trust among participants, as well as getting them to recall their feelings, thoughts, and experiences.

*Participants*. A total of 38 participants took part in the focus groups, distributed as follows: FG1: 7 schoolchildren (4 boys and 3 girls; M age = 15.71 years, SD = 1.98); FG2: 7 schoolchildren (1 boy and 6 girls; M age = 14.00 years, SD = 1.52); FG3: 8 parents (3 males and 5 females; M age = 46.88 years, SD = 1.89); FG4: 8 parents (3 males and 5 females; M age = 43.62 years, SD = 2.26); FG5: 5 PE teachers (4 males and 1 female; M age = 45.00 years, SD = 3.00).

*Procedure*. All focus group were conducted on 14th April 2019 and were held in a relaxed and calm environment at the rooms of the Viana High-Performance Surfing Center in Viana do Castelo. The sessions in each FG were conducted by 2 researchers. At the beginning of the session, the participants were reminded about the aim of the study and about data confidentiality and processing issues. They then read and signed the informed consent documents. All focus groups were tape-recorded and transcribed verbatim. To analyze the content and ensure transcription accuracy, the full transcription with respect to the audio file was randomly compared. Participants from the different FGs were identified with a capital letter each as follows: school children (S), parents (P), and teachers (T). In addition, a number was added to differentiate among them. All qualitative data were analyzed in the NVivo software version 12.0 for MAC (QRS International, Melbourne, Australia). A first theory-based codification proposal was designed, grounded on a previous systematic review on the field [8]. Data were deductively organized in the defined categories. The resultant initial structured data were read again, and new codes were created by means of an inductive analysis. Data were then reviewed and grouped into seven categories: (1) changes in the overall health; (2) physical activity; (3) mental health and well-being; (4) educational skills; (5) sense of community; (6) social behavior; and (7) environmental awareness.

## 3. Results

### 3.1. Changes in the Overall Health

Participants were asked about their current overall health perception and the changes that they felt they had experienced after their participation in the School Nautical Activities program. As is shown in Table 1, more than a half of the sample affirmed that their overall health was about the same after the project (54.3%, *n* = 323) but, additionally, more than 40% stated that their health was much better now (13.4%, *n* = 79) or somewhat better now (29.9%, *n* = 177) due to their participation in the program. The assessment of an improvement in their overall health was confirmed by most of the participants in the focus groups: “In general, I got healthier because we became very healthy outdoors” (S2); “the practice of surf gives me more health in general” (S1); and not only by children but also by their parents: “they spend a lot of time with their mobile phones, and it is also a way to get them out of their mobile phones and other technologies and make them healthier” (P7), and by their PE teachers: “the physical part, the improvement of the physical part, which is physical fitness in general terms. I thought it was also an important factor for them in particular” (T5).

### 3.2. Changes in the Physical Activity

The following table (Table 2) shows the difference between the frequency, duration, and intensity of the level of physical activity of the participants before the start of the school nautical activities and after the project, referring to the level of physical activity outside of the school PE lessons.

Although there existed a decrease in the number of participants who affirmed to participate 5 or more times a week (from 13.6%, *n* = 80, to 11.4%, *n* = 67) and 4 to 3 times a week (from 22.9%, *n* = 136, to 19.3%, *n* = 114), the number of schoolchildren who never undertook any physical activity was reduced from 14.6% (*n* = 86) to 2.5% (*n* = 14) after the project. Most of the participants from the diverse focus groups stated the importance of the project for the promotion of physical activity in children: “it helps us to maintain a physical activity, we distract, because life is not just a school life, in our case, to keep us active” (A6). Both teachers: “to promote physical activity. I think this has been one of the main objectives” (T1); “The clear increase in practitioners that this promotes. It sensitizes children who want to try it and want to return. If not, they would not come” (T4); and parents: “it is an excellent initiative to put them in contact with several modalities, even so that they can have the power of decision to say what they want, is the best way to know if they like it or not and to go on with it” (P6); agreed in declaring this clear increase in the degree of regular physical activity of the participants in the project after their participation in the school nautical activities.

Results in relation to the overall assessment (Table 3) of the effects on the frequency, duration, and intensity of the physical activity of the participants after their participation in the program are shown in Table 3. More than 70% admitted to have increased their degree of participation in physical activity very significantly (8.6%, *n* = 51), significantly (28.4%, *n* = 168), or moderately (37.3%, *n* = 221). It is important to take into account that it only refers to the physical activity undertaken outside of the PE sessions and the nautical activities within the program.

### 3.3. Mental Health and Well-Being

With respect to mental health and well-being, participants were asked to assess their feelings and thoughts in relation to the different statements shown in Table 4 after their participation in school nautical activities.

Results show that values for the answer “at no time” on any of the statements were minimal: from 5.4 (*n* = 32) to 2.2% (*n* = 13) of the participants; meaning that this was recorded by less than 30 participants from the total respondents. In all the items, the addition between the answers “all of the time” and “most of the time” exceeded 50%. The most noted results were for the statements “I feel active and vigorous” and “I feel interested in other people and new things.”

One of the things that participants valued the most in relation to mental health and well-being was the feeling of happiness when they were participating in these kind of activities: “Surf is happiness, because when I’m surfing, I feel well” (A7); “When surfing I’m happier, more relaxed” (A10). Furthermore, at the same time, most of the participants related this feeling of happiness with the freedom experienced when participating: “when you practice these sports, you feel freer, happier” (A9); “in general, I have happiness when I’m in contact with nature, and freedom” (A12). Furthermore, the improvement of other well-being factors, such as self-esteem, was also mentioned: “it improves their self-esteem, because it also teaches them to overcome fears and difficulties, and to go beyond what they thought” (P6); or calm and tranquility: “I get spiritual peace, because when I sail I feel calm and well with myself” (A7); “Surfing for them is synonymous of well-being, it is something that really does them good, contact with the sea, without a doubt. They feel great with surfing” (P5).

If we focus on the overall assessment (Table 5) of the effects on participants’ mental health and well-being, as shown in Table 5, the tendency observed in the previous statements was confirmed. The majority of the participants stated that their participation in school nautical activities had improved their mental health and perception of well-being: more than 70% considered that it to be very significant (10.4%, *n* = 61), significant (32.9%, *n* = 195), or moderate (31.1%, *n* = 185).

### 3.4. Education and Learning

In regard to education and learning, participants were asked to express their degree of agreement to the statements shown in Table 6, after their participation in the program activities.

As results show, more than 70% of the participants agreed or strongly agreed that they had learned to take care of their equipment, over 65% to interpret nature, and over 56% about climate conditions and meteorology. In the case of having improved their time management, the percentage decreased down to just under 45%, which is still a considerable result.

Most of the participants outlined their acquisition of healthy habits. That fact was verified by their parents and teachers: “I think that their care towards food has changed towards a healthy diet” (P5); “there were children who did not have autonomy, who did not dress nor undress, who did not wash the board… all this has changed; it creates routines and habits of those who begin to be aware” (T3). Participants also affirmed that, after their participation in school activities, their team spirit and their abilities for teamwork had improved: “in rowing, I value the teamwork. Because there is a lot of people, so if we don’t have the tune, it doesn’t work” (A7); “synchronization in the group… because it makes no sense to be in the same boat as a partner and you are rowing forward and your partner back” (A5).

Focusing on the overall assessment (Table 7) of the changes perceived by participants on their educational skills, the tendency of the concrete previous statements was also confirmed. A great majority (89.6%, *n* = 533) of the participants stated that School Nautical Activities had improved their learning, and only 9.1% (n = 54) indicated that their education had not been affected at all.

### 3.5. Sense of Community

The study also examined the sense of community and social commitment of the participants after their participation in the nautical activities program. Likewise, participants were asked to express their degree of agreement to the statements shown in Table 8.

Most of the participants agreed in saying that the nautical activities had provided them with some environmental values and social commitments that could be transferred to their daily life:

The promotion of active citizenship, the question of beach cleaning, the question of protecting the oceans from the issue now that it is fashionable, has appeared from plastics, but that has been a problem for a long time, there is every time more children involved in this because they are called to reason, with an active participation in these things (T1).

One of the most noted results was in relation to participants’ concern about what happened in the marine environment around them: almost 60% of the participants agreed or strongly agreed with that statement. That fact is evidenced by some of the feedback from the focus groups: “They see the dirty river, the dirty sea, they see the plastic on the beach, and they think: this is not only in the news, this happens, let’s do something. The spirit of community” (T3).

The results of the overall assessment (Table 9) of the changes in participants’ sense of community showed that only 6.6% (*n* = 39) of the participants considered that the project had not improved their citizenship and community activity or that they had insignificantly been modified (15.1%, *n* = 89). Quite the opposite, more than 70% stated that they had improved very significantly (10.8%, *n* = 64), significantly (31.8%, *n* = 189), or moderately (33.3%, *n* = 198).

The most valued statements were as follows: “I feel more connected to my school” and “I feel more concerned about what happens around me.” According to the statements of some of the participants:

“Respect is fundamental and that is what I learned by these sports. Because they are not only dependent on nature, but also on other people; and being in contact with others is always necessary to have respect for everyone, so that everything goes well” (S4).

### 3.6. Social Behavior

In the same way, participants were asked to express their degree of agreement to the statements shown in Table 10, in relation to the changes perceived in their social behavior, after their participation in the program activities.

Social relationships and friendship were outlined by the majority of the participants as one of the most noted outcomes of the project: “living these activities with other people makes me happy, it’s better to go out and do sports with friends than to be alone” (A13); “It is really fun being with friends and it is better if it is practicing these sports” (A5). They also outlined that nautical activities allowed them to establish new relationships: “it has created new friendships” (P2); “I think that this experience will *mark* them… because it has stablished new friends’ (P8); “they have been with friends different from the usual ones” (T2).

As has occurred in previous factors, results of the overall assessment (Table 11) of the effects on social behavior demonstrated that most of the participants affirmed that it had been improved after the project very significantly (10.9%, *n* = 64), significantly (29.9%, *n* = 177), or moderately (32.9%, *n* = 195); and, on the contrary, 25.1% (*n* = 149) considered that it had been insignificantly (16.5%, *n* = 98) modified or that it had not been improved at all (8.6%, *n* = 51). In that case, the most rated statements were as follows: “I am more respectful with the marine environment” and “I am more respectful with the people around me.”

### 3.7. Environmental Awareness

The last factor analyzed was the degree of environmental awareness generated after the participation in the School Nautical Activities program. Participants expressed their degree of agreement to the statements shown in Table 12, in relation to the effects that they thought to have experienced in relation to their environmental consciousness, after their participation in the project. 

Results show that more than 80% of the participants agreed that, after the project, ‘being in the sea makes them feel refreshed and revitalized’ or that ‘they are concerned about damage to the marine environment’.

The majority of the children participating in the focus group affirmed that their environmental awareness had increased after the project:

“We protect nature, we will not litter the sea knowing that we surf it; we naturally won’t do it” (A1).

“It is not good to contaminate the place where we train. We are closer to an environment, we automatically feel a sense of protection, of what is ours” (A3).

This change was also underlined by parents: “I think my son shows that he is more respectful of the environment” (P8); “a great communion with nature and learning values of respect for her; she already knew it from the Scouts but in surfing she has increased it” (P3); and teachers: “they also learn to respect nature and promote coexistence and friendship between them, but also knowledge of the conditions of the sea and the river, depending on the modality they are learning” (T4) / “the contact with nature awakens environmental awareness in many children who had never thought about it” (T3).

If we focus on the overall assessment (Table 13) of this factor, we can confirm the tendency observed in the concrete previous statements. The majority of the participants affirmed that school nautical activities had improved their environmental awareness and their feelings toward the marine environment very significantly (21.0%, *n* = 124), significantly (32.1%, *n* = 190), or moderately (29.7%, *n* = 176).

As has been evidenced by the individual analysis of the results for each factor, benefits emerged in all of them. Thus, as shown in Table 14, there exists a positive and moderate or large significant correlation between all the analyzed factors.

Additionally, correlations between participants’ global valuation and the project with the benefits on the different factors analyzed is shown in Table 15. Participants were asked to state how much they would be willing to spend on continuing to take part in the project if they were only getting 10 euros every week from their parents. As Table 15 shows, there exists a small significant correlation between this global valuation and the benefits experienced in all the analyzed factors.

## 4. Discussion

This study has attempted to determine the effects of a School Nautical Activities program on the health of the participants and the degree to which the participation in outdoor sports in blue spaces generated benefits in the different dimensions of their well-being.

In general terms, several previous studies had provided evidence about the positive relationship between the practice of outdoor sports and health benefits [1,2,3,8]. Our study, although it is based in a school program and participants’ perception, has taken a step forward and has given exact figures on the effects on the overall health, showing that more than 40% affirmed to have experienced benefits from it and a better quality of life, coinciding with the findings in previous works [9,24].

Considering physical activity, the number of schoolchildren who never participated in any physical activity was reduced after the project, demonstrating that physical activity initiatives in coastal areas may encourage and facilitate being more active [10]. Furthermore, several benefits were found in relation to mental health and well-being, as already stated in numerous preceding studies [15,16,25]. Concretely, for example, this has been highlighted in terms of stress management [9], cognition and concentration [11], or good spirits and motivation [13]. Participants highlighted an increase in their sensation of calmness and tranquility, which contrasts with the results of other studies that state that such feelings are decreased following outdoor exercise [10]. However, our data were based on a series of interventions over time and not on a single episode of activity, and they were also mediated by the mixed methodology used, and so we have more data to conduct the analyses. Furthermore, being enrolled only in a marine environment may also have impacted and influenced participants’ sense of well-being.

In relation to education and life-long learning, improvements in knowledge and habits of behavior were evidenced in our study, along the same lines as in related studies [14]. Previous studies coincide with our findings on the increase in educational performance [12] or the improvements in capacity of attention and interest in school studies [26]. In this particular case, one of the mediators may also be the inclusion and support for those children with disabilities in the same classes, which helps to reduce stereotypes, while empowering participants [17]. Participants in our study recognized that their participation in the project had facilitated the acquisition of some values and social commitments transferable to their daily life, as stated in related studies [27,28]. In fact, previous works concur with our findings on the benefits in the sense of community and social behavior of performing of outdoor activities being more concerned about what happens around them and respectful with people around and empowering participants [17].

Our results, in accordance with evidence from previous works [29,30,31], also show an improvement in the environmental awareness of the schoolchildren after their participation in the project. Even so, previous statements did not find enough evidence on the causality of better environmental behavior [32]. Again, these different directions may be mediated with the findings of our study being on “blue spaces” activities, reinforcing a previous work focused on marine-based environments [33].

The results obtained both in this study and in those mentioned heretofore seem to regard the participation in outdoor sports as considerably significant for the generation of health and social benefits. However, we would like to put that statement into context and to point out that this relationship is produced only when a set of favorable conditions for the participation are guaranteed and that it does not exclusively depend on the physical activity.

Furthermore, our study did not reveal any negative effects from the participation in outdoor sports on the health and well-being of participants as has appeared in other studies. For example, in the increase of the risk of some illnesses [34], the increment of some injuries associated with some specific sports [35], or the appearance of potential social conflicts because of the cohabitation of different sports activities in the same natural area [29,36].

## 5. Conclusions

This work has provided new empirical evidence on the positive relation between participating in physical activity in outdoor (and especially blue) spaces and human health and well-being. However, much more research is needed in this field. This work has shed some light on the existence of a positive relationship of regular participation for children and adolescents. Results have not only shown that regular participation in nautical sports such as surfing, rowing, sailing, and canoeing in blue spaces generates benefits on the physical, mental, educational, and social dimensions of children and adolescents but also that there exists a significant association between the effects on these dimensions. Moreover, these results may also have some practical implications in the design of physical education curriculum and promoting political decisions and investments to facilitate nautical activities at schools.

This study is impaired by several limitations that must be considered when highlighting our results. One such limitation is the fact that the survey was administered at the same moment to all the respondents independent of the number of school years in which they had participated in the project or the time in which they had finalized their participation. Another limitation is that data were not gathered right after the participation in the program. This fact may hinder the statement that the well-being improvements found in the group of participants were actually promoted by their participation in the program, because these feelings could have also been generated by a retrospective return to the memories of that period. In addition, sample was limited to the participants in the program, so results could not be compared with the ones of a control group. Another limitation that must be outlined is that the assessment of health and social benefits has only been based on participants’ self-perception and no anthropometric or previous physical condition or mental state indicators were considered.

These limitations highlight a compelling opportunity for future studies, which should shed additional light on this topic. A longitudinal study focusing on a group of schoolchildren could be designed with a detailed analysis of the effects generated at the end of each school year by their participation in the project. This study would therefore allow us to engage in a real-time monitoring process from the beginning of the project and following-up the analysis in the subsequent years with the aim of analyzing the lasting of the effects throughout time after the program was ended.

To conclude, these data provide support for the idea that there are clear benefits for children’s health of participating in sports in outdoors settings, especially in blue spaces. This information may be used in the definition of educational and health promotion strategies in the development of social behavior, sense of community, educational skills, and environmental protection programs for children and adolescents of school age. However, only through additional research shall we be able to fully verify this relationship and to develop outdoor sports programs that could bring significant social and health benefits to society.

## Figures and Tables

**Table 1:** General health during and after the project.

**General Health during the Project**					
Very Good	Good	Fair	Bad	Very Bad	Missing
47.2	43.9	7.2	0.8	0.2	0.7
**General health after the project**					
Much better now	Somewhat better now	About the same	Somewhat worse now	Missing	
13.4	29.9	54.3	1.2	1.2	

**Table 2:** Changes in the amount physical activity before and during the project.

**Physical Activity before the Project, in General**					
5 or more Times a Week	4 to 3 Times a Week	Twice a Week	Once a Week	Never	Missing
13.6	22.9	32.8	14.8	14.6	1.3
More than 120 min	>90–120 min	>60–90 min	>30–60 min	Less than 30 min	Missing
11.9	18.8	30.4	18.3	6.9	13.6
Easy	Moderate	Vigorous	Missing		
22.9	53.3	10.8	13.1		
**Physical Activity during the Project, Out-Of-School**					
5 or more Times a Week	4 to 3 Times a Week	Twice a Week	Once a Week	Never	
11.4	19.3	26.6	19.5	2.5	
More than 120 min	>90–120 min	>60–90 min	>30–60 min	Less than 30 min	Missing
15.6	16.0	26.2	14.8	5.2	22.2
Easy	Moderate	Vigorous	System		
20.5	47.1	10.4	22.0		

**Table 3:** Overall assessment of the effects on physical activity after the project.

Effects on Physical Activity—Overall Assessment					
Very Significant	Significant	Moderate	Insignificant	Not at All	Missing
8.6	28.4	37.3	13.9	7.9	2.9

**Table 4:** Mental health and well-being after the project.

	Feelings on Mental Health and Well-Being after the Project						
**Feelings Statements**	All of the Time	Most of the Time	More than Half of the Time	Less than Half of the Time	Some of the Time	At no Time	Missing
I feel cheerful and in good spirits	27.9	41.3	13.8	2.5	10.6	2.2	1.7
I feel calm and relaxed	21.7	37.5	17.5	6.6	11.4	4.0	1.3
I feel active and vigorous	30.1	37.5	15.3	5.2	7.6	3.0	1.3
I wake up feeling fresh and rested	14.5	30.1	20.7	11.4	12.1	9.9	1.3
My daily life is filled with things that interest me	19.8	37.1	20.7	7.7	9.9	3.4	1.3
I feel interested in other people and new things	31.3	35.6	16.5	4.5	7.2	3.7	1.2
I think clearly	15.6	39.8	20.7	7.4	9.6	5.5	1.3
I feel confident	29.6	31.1	15.8	6.1	11.1	5.4	1.0

**Table 5:** Overall assessment of the effects on mental health and well-being.

Effects on Mental Health and Well-Being—Overall Assessment					
Very Significant	Significant	Moderate	Insignificant	Not at All	Missing
10.4	32.9	31.1	15.3	8.1	2.2

**Table 6:** Education and learning after the project.

Educational Skills Developed after the Project						
**Skills Statements**	Strongly Agree	Agree	Neither Agree nor Disagree	Disagree	Strongly Disagree	Missing
I have learned to take care of the equipment	34.8	46.6	13.1	2.0	1.7	1.8
I have learned to interpret nature	22.4	43.7	19.2	8.1	4.9	1.8
I have learned about climate conditions and meteorology	17.6	39.8	26.4	8.2	6.2	1.7
I have improved in my time management	17.1	27.7	34.1	10.6	8.4	2.0

**Table 7:** Overall assessment of the effects on educational skills.

Effects on Educational Skills—Overall Assessment					
Very Significant	Significant	Moderate	Insignificant	Not at All	Missing
9.1	28.1	36.6	15.8	9.1	1.3

**Table 8:** Sense of community after the project.

Sense of Community after the Project						
**Sense of Community Statements**	Strongly Agree	Agree	Neither Agree nor Disagree	Disagree	Strongly Disagree	Missing
I make more decisions by myself	17.1	40.5	28.1	8.1	4.9	1.3
I have made new friends	21.8	24.4	22.0	17.6	12.4	1.7
I have better relationships with my friends, family, and teachers	21.0	37.0	27.2	8.2	5.0	1.5
I am more trusting of people around me	20.8	36.0	26.1	8.7	6.6	1.8
I feel more concerned about what happens around me	24.5	35.0	26.6	7.1	5.0	1.8

**Table 9:** Overall assessment of the effects on the Sense of community.

Sense of Community—Overall Assessment					
Very Significant	Significant	Moderate	Insignificant	Not at All	Missing
10.8	31.8	33.3	15.1	6.6	2.5

**Table 10:** Social behavior after the project.

Social Behavior after the Project						
**Social Behavior Statements**	Strongly Agree	Agree	Neither Agree nor Disagree	Disagree	Strongly Disagree	Missing
I am more respectful with the people around me	27.6	42.2	20.2	5.2	3.7	1.2
I take more care of my stuff	26.7	40.0	21.3	5.5	4.9	1.5
I participate more actively in classroom activities	15.3	33.6	32.4	9.7	7.6	1.3
I share my stuff more with others	18.8	36.5	27.4	9.2	6.9	1.2

**Table 11:** Overall assessment of the effects on the social behavior.

Social Behavior—Overall Assessment					
Very Significant	Significant	Moderate	Insignificant	Not at All	Missing
10.9	29.9	32.9	16.5	8.6	1.2

**Table 12:** Environmental awareness after the project.

Environmental Awareness after the Project						
**Environmental Awareness Statements**	Strongly Agree	Agree	Neither Agree nor Disagree	Disagree	Strongly Disagree	Missing
Being in the sea makes me feel refreshed and revitalized	46.7	36.3	12.1	2.4	1.3	1.2
Spending time out in the sea is an important part of my life	31.4	31.3	25.5	6.9	3.9	1.0
I am concerned about damage to the marine environment	44.5	36.5	13.3	3.4	1.3	1.0
Having open blue spaces close to where I live is important for me	37.5	32.6	20.5	4.5	3.2	1.7

**Table 13:** Environmental awareness. Overall assessment.

Environmental Awareness—Overall Assessment					
Very Significant	Significant	Moderate	Insignificant	Not at All	Missing
21.0	32.1	29.7	11.1	4.7	1.3

**Table 14:** Correlations between benefits on different factors.

Correlations between Benefits on Different Factors						
		(1)	(2)	(3)	(4)	(5)
(1) Mental health and well-being	Spearman’s rho	1				
	N	590				
(2) Educational skills	Spearman’s rho	0.49 **	1			
	N	585	587			
(3) Sense of community	Spearman’s rho	0.49 **	0.64 **	1		
	Sig. (2-tailed)	0.00	0.00			
	N	587	584	588		
(4) Social behavior	Spearman’s rho	0.47 **	0.66 **	0.74 **	1	
	N	587	584	586	589	
(5) Environmental awareness	Spearman’s rho	0.42 **	0.50 **	0.46 **	0.54 **	1
	N	587	584	585	586	589

** Correlation is significant at the 0.01 level (2-tailed).

**Table 15:** Correlations between global valuation of the project and benefits on different factors.

Correlations between Global Valuation of the Project and Benefits on Different Factors						
		(1)	(2)	(3)	(4)	(5)
Global valuation of the project (1–10)	Spearman’s rho	0.21 **	0.30 **	0.26 **	0.27 **	0.19 **
	N	570	566	568	569	570

** Correlation is significant at the 0.01 level (2-tailed).
